# EANM expert opinion: How can lessons from radiobiology be applied to the design of clinical trials? Part I: back to the basics of absorbed dose–response and threshold absorbed doses

**DOI:** 10.1007/s00259-024-06963-9

**Published:** 2024-11-12

**Authors:** Jean-Pierre Pouget, Pablo Minguez Gabina, Ken Herrmann, Desirée Deandreis, Mark Konijnenberg, David Taieb, Fijs W. B. van Leeuwen, Jens Kurth, Uta Eberlein, Michael Lassmann, Katharina Lückerath, For the EANM Radiobiology Working Group

**Affiliations:** 1 Institut de Recherche en Cancérologie de Montpellier (IRCM), INSERM U1194, Université de Montpellier, Institut Régional du Cancer de Montpellier (ICM), Montpellier, France. Equipe Labellisée Ligue Contre Le Cancer, INSERM U1194/IRCM, 208 Rue Des Apothicaires, 34298 Montpellier, France; 2 Department of Medical Physics and Radiation Protection, Gurutzeta-Cruces University Hospital/Biocruces Health Research Institute, Barakaldo, Spain; 3 Department of Nuclear Medicine, University of Duisburg-Essen and German Cancer Consortium (DKTK)-University Hospital Essen, Hufelandstr. 55, 45147 Essen, Germany; 4 Nuclear Medicine and Endocrine Oncology, Gustave Roussy and Paris Saclay University, 114 Rue Edouard Vaillant, Villejuif, France; 5 Radiology & Nuclear Medicine Department, Erasmus MC, Rotterdam, The Netherlands; 6 Nuclear Medicine Diagnostic Imaging and Endoradiotherapy Center Aix-Marseille University CHU de La Timone, Marseille Cedex 5, Marseille, France; 7 Interventional Molecular Imaging Laboratory, Leiden University Medical Centre, Leiden, the Netherlands; 8 Department of Nuclear Medicine, University Medical Center Rostock, Rostock, Germany; 9 Department of Nuclear Medicine, University of Würzburg, Würzburg, Germany

**Keywords:** Radiopharmaceuticals, Radiobiology, Biomarkers, Dosimetry, Radiopharmaceutical therapy

## Abstract

**Purpose:**

This study by the EANM radiobiology working group aims to analyze the efficacy and toxicity of targeted radionuclide therapy (TRT) using radiopharmaceuticals approved by the EMA and FDA for neuroendocrine tumors and prostate cancer. It seeks to understand the correlation between physical parameters such as absorbed dose and TRT outcomes, alongside other biological factors.

**Methods:**

We reviewed clinical studies on TRT, focusing on the relationship between physical parameters and treatment outcomes, and applying basic radiobiological principles to radiopharmaceutical therapy to identify key factors affecting therapeutic success.

**Results:**

The analysis revealed that mean absorbed dose alone is insufficient to predict treatment response or toxicity. For absorbed doses below a certain threshold, outcomes are unpredictable, while doses above this threshold improve the likelihood of biological responses. However, even at higher absorbed doses, response plateaus indicate the need for additional parameters to explain outcome variability, including heterogeneity in target expression, anatomical disease location, (epi)genetics, DNA repair capacity, and the tumor microenvironment, aspects that will be discussed in Part II of this analysis.

**Conclusion:**

Understanding radiobiology is crucial for optimizing TRT. More dosimetric data is needed to refine treatment protocols. While absorbed dose is critical, it alone does not determine TRT outcomes. Future research should integrate biological parameters with physical dosimetry to enhance efficacy and minimize toxicity.

## Introduction

Despite its long history, targeted radionuclide therapy (TRT) has only recently attracted attention as a main contributor to cancer therapy. The recent USA Food and Drug Administration and European Medicines Agency approval of two new innovative TRTs was based on phase 3 clinical trial data that demonstrated significant improvement in progression-free survival (PFS) with [^177^Lu]Lu-DOTA-TATE (Lutathera™) in patients with somatostatin receptor (SSTR)-expressing neuroendocrine tumours (NETs; NETTER-1 and NETTER-2 trials) [1, 2], and in overall survival (OS) and PFS with [^177^Lu]Lu-PSMA-617 (Pluvicto™) in patients with prostate-specific membrane antigen (PSMA)-expressing metastatic castration-resistant prostate cancer (mCRPC; VISION trial) [3]. After this success, many TRTs based on new targets, radionuclides, and platforms are currently in (pre)clinical development [4].

The phase 3 trials included the selection of patients who may benefit from TRT by imaging to confirm target expression. The VISION study showed clinically relevant hazard ratios (HR) of 0.40 (95% CI, 0.29–0.57) for PFS and 0.62 (95% CI, 0.52–0.74) for OS in favour of TRT with [^177^Lu]Lu-PSMA-617. However, half of the patients did not respond to TRT: reduction of prostate-specific antigen (PSA; a tumour marker) was < 50% [3]. [^177^Lu]Lu-DOTA-TATE treatment resulted in a HR of 0.18 (95% CI, 0.11–0.29) for PFS in favour of TRT, but OS was not prolonged significantly (HR 0.84 [95%CI, 0.60–1.17], two-sided *p* = 0.30) [1, 5]. The phase III NETTER-2 study demonstrated efficacy of TRT as first-line treatment in patients with Grade (G) 2 and G3 advanced gastroenteropancreatic neuroendocrine tumours [2]. However, to increase response rates and the survival benefit for patients in the future, it is imperative to understand why a substantial group of patients does not respond to TRT despite target expression, as defined by pre-therapeutic imaging.

The key to understanding the mechanisms underlying response or non-response to TRT lies in deciphering the radiobiology of tumours. The European Association of Nuclear Medicine (EANM) has established a radiobiology working group to analyse these aspects [6, 7]. The aim of this paper was to provide answers and describe the lessons learnt from past clinical trials from a radiobiological perspective. The present Part I focuses on the relationship between physical parameters and the outcome of TRT, while the following Part II will focus on tumour biology as the link between absorbed dose and efficacy.

## Basics on the radiobiology of tumours and normal tissues

### Basics

**Fig. 1:**
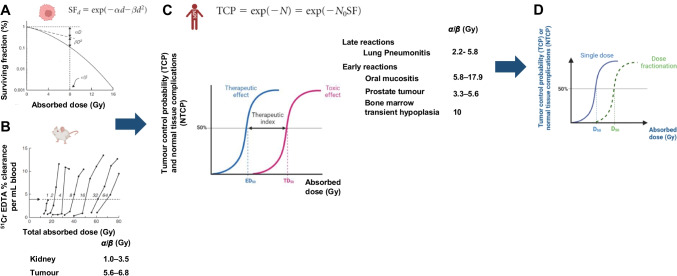
Schematic representation of determination of the α/β (Gy) parameters using **A)** The clonogenic survival fraction of irradiated cells, **B** Tissue response in animals. **C** Theoretical TCP and NTCP curves obtained in patients treated with EBRT based on the LQ model. Examples of α/β (Gy) are given (data from ([9–11]). D) Role of absorbed dose fractionation on TCP and NTCP

Radiobiology is the science that studies the biological effects of radiation. In conventional external beam radiotherapy (EBRT), it is considered that most biological effects (e.g., efficacy and toxicity) depend, in large parts, on the absorbed dose delivered to the tumour and normal tissue, leading to cell death. Cell death is induced when the radiation-induced damage and cell stress exceed the cell’s capacity to repair the damage. Cell death contributes to tissue dysfunction when it leads to the inactivation of the functional subunits (FSU) that organise tissues [8]. This is clinically observed when there is an imbalance between cell renewal and cell depletion. Tissue dysfunctions can be deterministic (non-stochastic) radiation effects if they occur in all irradiated persons (generally, days to months after irradiation). Conversely, stochastic effects include the development of radiation-induced cancer. They occur late after exposure (years) and only in a subgroup of irradiated persons. In cancer therapy, deterministic effects are sought to control tumours and are feared due to normal tissue complications (e.g., bone marrow, kidney, and salivary gland toxicities). The deterministic effects occur above a threshold absorbed dose of radiation. They occur days or weeks and up to months after exposure and their severity is proportional to the absorbed dose. A way to quantify the effect of radiotherapy on tumour or normal tissues is to determine the probability of tumour control (TCP) and the probability of normal tissue complications (NTCP). This implies defining a biological criterion for TCP and NTCP. Therefore, TCP and NTCP curves plot the occurrence of a specific biological endpoint as a function of the absorbed dose. TCP and NTCP are not linear functions of the absorbed dose but follow an S-shaped curve (sigmoid) (Fig. 1)*.*

### TCP and NTCP

The radiobiological interpretation of the sigmoid absorbed dose–response relationship (TCP and NTCP) is the clonogenic death of the target cells (i.e., clonogenic cells) that constitute the FSU [12] of the normal tissues or the tumour according to a linear quadratic (LQ) model (Fig. 1A). LQ models describe the cell and tissue response according to alpha and beta parameters related to the cell intrinsic radiosensitivity and the tissue repair capacities, respectively. The LQ model has been widely described in the literature [13]. Alpha and beta parameters can be determined from survival curves of cells in vitro (Fig. 1A), but also from data obtained in animals at the tissue scale (Fig. 1B), before being validated in patients (Fig. 1C). The target cell theory states that the response at the tissue level is solely due to the death of the key clonogenic cells making up the tissue [14]. However, this theory is no longer valid, particularly concerning the late effects of radiation (e.g., kidney damage, lung fibrosis), where tissue remodelling involves a complex interplay of damage to various cell populations (organ parenchyma, connective tissue, capillaries, immune cells) [15].

### Role of the total absorbed dose, fractionation, and treatment duration

It took about 40 years (~ 1900-1940s) for EBRT scientists to empirically arrive at the irradiation scheme we know today. This scheme aims to improve tumour control and reduce complications in normal tissue. In this scheme, a total initial absorbed dose (D, in Gy) is divided into several fractions, each of which delivers an absorbed dose d and is spread over a given period of time (treatment duration). Fractionation of the total absorbed EBRT dose into several fractions allows the repair of sublethal DNA lesions and protects late responding (and slowly proliferating) tissues, while early responding tissues are more sensitive to treatment duration (i.e., to the time between first and last absorbed dose), which favours cell proliferation [16]. It is tempting to apply the principles of EBRT to TRT. However, major differences exist. Specifically, TRT is low absorbed dose rate irradiation, results in a heterogeneous absorbed dose distribution in a tumour lesion, and remains within tumour cells for hours to days [17, 18]. In addition, TRT uses repeated injections (cycles), each of which delivers a dose (which decreases between cycles) and contributes to the final total absorbed dose. Unlike EBRT, TRT does not start from a situation where an initial absorbed dose leading to a biological endpoint is divided into several sub-absorbed doses to avoid toxicity and the activities injected are not modified between cycles. Consequently, the repetition of cycles does not correspond strictly to absorbed dose fractionation and therefore, the effect of retreatment on the TCP and NTCP curves is different from that of fractionation (Fig. 1D).

## Clinical lessons from dosimetry: tumour control probability

### Threshold absorbed dose for tumour control probability

Sigmoid TCP and NTCP curves are still used for treatment planning in EBRT [14, 19]. These curves allow predicting the biological effect from a given absorbed dose. In practice, EBRT absorbed doses are always above the threshold absorbed dose required for a given anti-tumour effect (%TCP); the radiation oncologist must then find a balance between the appropriate TCP value and the acceptable NTCP value. The medical physicist designs a patient-tailored treatment plan complying to these constraints.

The establishment of TCP and NTCP curves in the field of TRT is not straightforward and is still the subject of debates for the following reasons: **i)** there is generally a lack of information on absorbed dose–effect correlations as absorbed doses are not routinely calculated in TRT; **ii)** the irradiation pattern is completely different in TRT and EBRT, as TRT delivers a continuous low absorbed dose rate irradiation (radiation delivery per unit time, < 1 Gy.h^−1^), while EBRT delivers acute irradiation at more than 1000-fold higher dose rate (2 Gy.min^−1^) [17, 20]. The lower absorbed dose rate of TRT favours tissue or lesion repair and higher absorbed doses are required to observe a given TCP or NTCP. Moreover, the longer treatment intervalls in TRT may favour cell proliferation (e.g., of tumour cells) [16, 21]*.* Within the LQ model, knowledge of alpha and beta values in TRT is less developed than in EBRT. As a first approximation the beta value could be neglected because cells have more time to repair their lesions due to the low absorbed dose rate [22, 23]. However, this is not always observed [24] and more studies are needed to deepen on the knowledge of alpha and beta values in TRT; **iii)** EBRT treats predefined localised single or oligometastatic tumours, while TRT can treat metastatic disease with multiple tumour sites for which absorbed doses and absorbed dose distribution may vary and cannot be predefined. The absorbed dose(s) delivered during TRT depend(s) on the administered activity, on the biological, chemical and physical characteristics of the radiopharmaceutical and on patient-specific factors, such as tumour target expression, tumour tissue composition, anatomical location of the lesion(s), and systemic factors, among others; and lastly, **iv)** the relationship between regression/progression of multiple lesions and patient outcome is more complex than when a single lesion is treated.

Before going any further, it should be considered that image-based dosimetry calculation in TRT is less standardised than in EBRT and the methodology of calculating the absorbed dose is still being improved. As a consequence, the calculated absorbed dose may vary from study to study, and the number of patients varies among studies. Therefore, the obtained absorbed dose values should be treated with caution, particularly when comparing studies [25, 26]. New data will be needed to validate or invalidate this interpretation and systemic meta-analyses on this topic are definitively needed.

Here, in the spirit of TCP, we have tried to correlate a biological criterion with the absorbed dose of irradiation delivered to the tumour. The first striking feature of TRT is the wide range of tumour absorbed doses (when absorbed doses are available): i) between 2 and 77 Gy per cycle of 7.4 GBq [^177^Lu]Lu-DOTA-TATE (*n* = 41, *n* = 37, *n* = 35, *n* = 90 patients with NETs at different locations) [27–30], ii) between 7.3 and 24.4 Gy from whole body assessment or 3.4–73.9 Gy/GBq in tumour-bearing bone for [^177^Lu]Lu-DOTA-PSMA (*n* = 30 patients with mCRPC) [31, 32], iii) between 0.6 and 44.1 Gy for [^223^Ra]Ra-Cl_2_ (*n* = 5 patients with mCRPC) [33] (reviewed in [34]).

Tumour volume is the first biological endpoint used to assess the effectiveness of TRT and can be defined using RECIST [based on the standardized uptake value (SUV)] or PERCIST criteria.

#### Hebert’s study

**Fig. 2:**
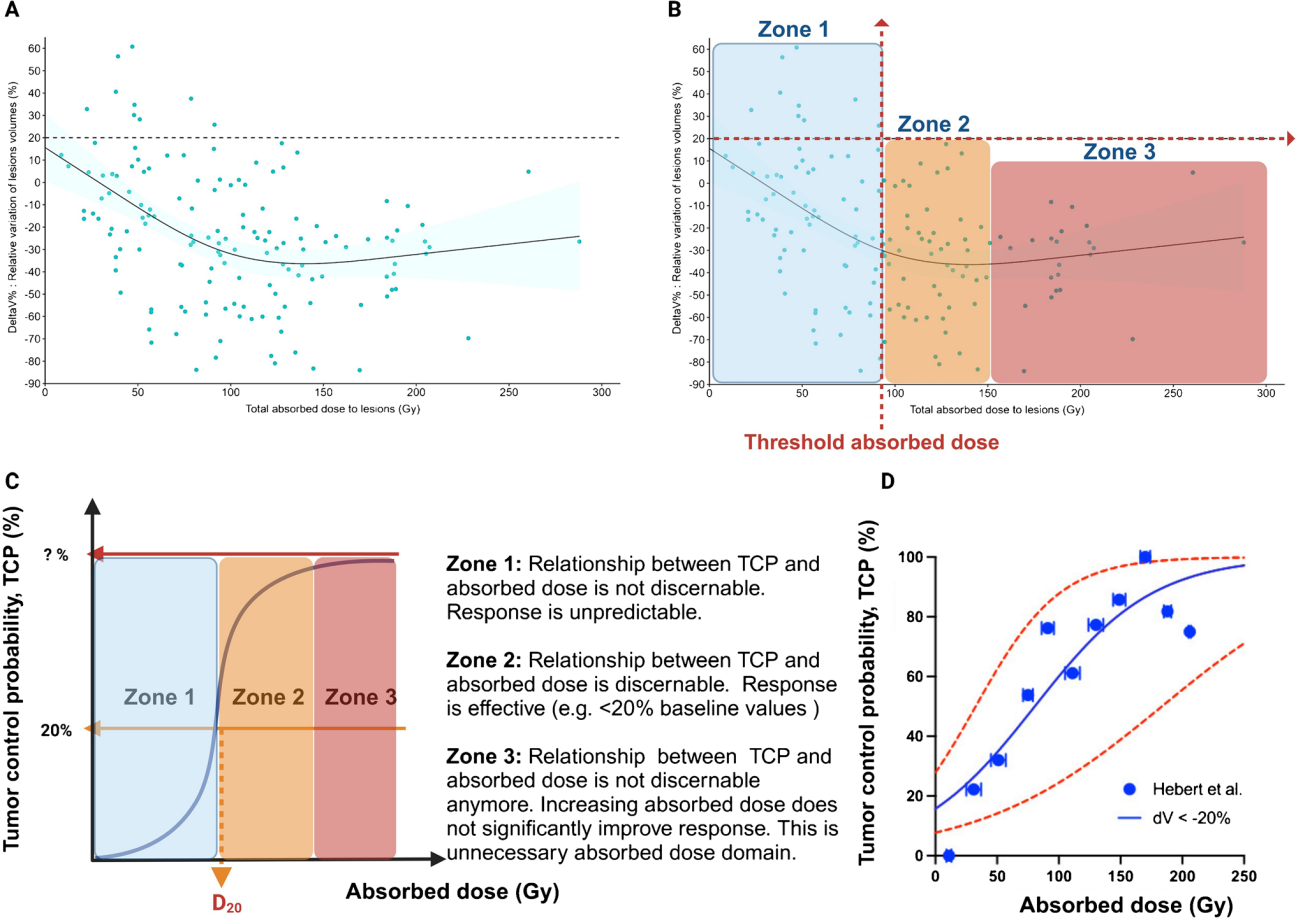
**A** Patients (*n* = 34 patients, *n* = 35 dosimetric datasets [re-challenged patient], *n* = 146 lesions) with NET were treated with 4 cycles of [^177^Lu]Lu-DOTA-TATE (7.4 GBq per cycle). The relative variation in lesion volume measured using CT (before and at the end of treatment) was expressed as a function of the cumulative lesion absorbed dose [29]. **B** We propose to define Zone 1 (solid line) corresponding to mean absorbed doses (< ~ 90-100 Gy) associated with an increase in volume > 20% from baseline in some lesions (unpredictable response). Zone 2 corresponds to mean absorbed doses (> ~ 100 Gy, threshold absorbed dose) associated, for all lesions, with an increase in volume < 20% (effective response). Zone 3 corresponds to the range of higher mean absorbed doses (> > ~ 175 Gy) with a variation in volume for most of lesions < 0% but relatively little improvement in treatment efficacy. This is the plateau zone, or the zone of unnecessary absorbed doses. The boundaries of zones 2 and 3 are arbitrary and depend on the biological criterion used (dashed lines) (adapted with permission from [29]). **C)** Definition of zones 1, 2 and 3. **D)** Data from Fig. 2A were grouped in dose cohorts and the probability of treatment success (i.e., a relative variation in lesions volume < 20%) was calculated. To this end the individual values in Fig. 2A were assigned to an absorbed dose cohort characterised by an average dose plotted on the x-axis in Fig. 2D. Within each cohort it was then possible to calculate the probability of a “success”, namely relative variation in lesions volume < 20%)

In the study by Hebert et al*.* [29], patients with NET (*n* = 34 patients, *n* = 35 dosimetric datasets [re-challenged patient], *n* = 146 lesions) were treated with 4 cycles of [^177^Lu]Lu-DOTA-TATE (7.6 GBq per cycle). Dosimetry was performed and the relative variation in lesion volume measured using CT (before and at the end of treatment) was expressed as a function of the cumulative lesion absorbed dose [29] (Fig. 2A). In a lesion based analysis, NET control (volume increase < 20% from baseline) was observed for mean absorbed doses above a threshold absorbed dose of 90–100 Gy after 4 cycles, while the highest lesion absorbed doses reached ~ 288 Gy. From Fig. 2A, we propose to define 3 zones. The unpredictable Zone 1 applies to mean absorbed doses below 90–100 Gy and includes some lesions with a volume progression > 20%. Zone 1 is called the “unpredictable response zone” (absorbed dose < threshold absorbed dose), since the variation in lesion volumes does not directly correlate with the absorbed dose and remains for some lesions above 20%. Zone 2 is called “effective response zone” as all lesions volume changes are less than 20% for all lesions (absorbed dose > threshold absorbed dose). Zone 3 is referred to as the "unnecessary absorbed dose zone" because the decrease in lesion volume is comparable to that observed in zone 2, indicating that the response might have reached a plateau (Fig. 2C).

An alternative way to rethink the traditional EBRT-TCP curve is to replace TCP with the probability of reaching an alternative endpoint. In Fig. 2D, the data shown in Fig. 2A are re-plotted to show the probability of an arbitrarily chosen 20% relative variation in lesion volume. This means that a lesion whose PET-volume variation is < 20% corresponds to 100% TCP. These plotted data suggest higher treatment effectiveness, with, for example, 50% and 90% probability of relative variation in lesion volume < 20%, at doses of about 75 Gy and 160 Gy, respectively. In this approach, using an averaged value can obscure data dispersion observed in Fig. 2A, although it could simplify clinical decision-making. It also incorrectly suggests a well-established dose–response relationship that allows predicting the efficacy of a given dose. In contrast we can say that above a threshold dose (e.g., 75 Gy or 160 Gy), the chosen probability of achieving a certain biological effect (50% and 90% probability of relative variation in lesion volume < 20%) is substantial.

#### Alipour’s study

**Fig. 3:**
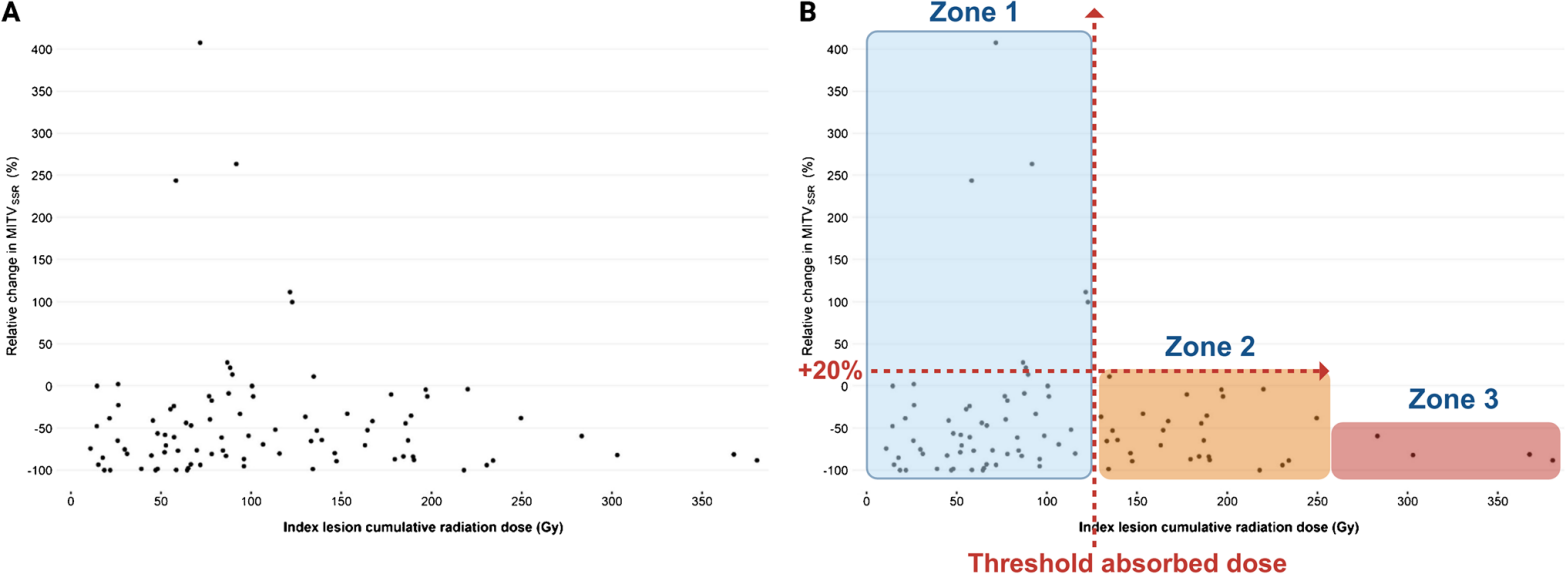
**A** Patients with NETs were treated with 2–5 cycles of [^177^Lu]Lu-DOTA-TATE (7.6 GBq per cycle). Relative change in the classifier “molecular imaging of tumour volume using SSTR detection” (MITV_SSTR_) with [^68^ Ga]Ga-DOTA-TATE was reported as a function of the cumulative lesion absorbed radiation dose. **B** Zone 1 (solid line) corresponds to absorbed doses associated with an increase in volume > 20% from baseline in some lesions (unpredictable response, absorbed dose < ~ 110-125 Gy). Zone 2 corresponds to absorbed doses (> ~ 110-125 Gy) associated with a decrease in size or an increase in size < 20% for all lesions (effective response). Zone 3 corresponds to the range of higher absorbed doses (> > ~ 110-125 Gy) that meet the criteria for zone 2 but offer relatively little improvement in treatment efficacy. This is the plateau zone, or the zone of unnecessary absorbed doses. The boundaries of zone 2 and zone 3 are arbitrary and depend on the biological criterion used (dashed lines) (adapted with permission from [30])

The second example describes 90 patients with NET who were treated with 2–5 cycles of [^177^Lu]Lu-DOTA-TATE (7.6 GBq per cycle); 68% of the patients concomitantly received chemotherapy [30]. Response was assessed by molecular imaging of tumour volume (MITV) using PET/CT detection of SSTR (MITV_SSTR_). MITV_SSTR_ was reported as a function of the cumulative lesion radiation dose (Fig. 3A). It is important to note that the data available for most TRT studies to date reflect the mean absorbed dose to the tumour, which does not take into account the heterogeneous distribution of the radiopharmaceutical and, therefore, of the absorbed dose. Working with the mean tumour absorbed dose, we again propose to divide the tumour response into three zones (Figs. 3B). A threshold absorbed dose of ~ 125 Gy is required to observe a MITV_SSTR_ change close to 20%. The latter value of 20% was chosen based on the RECIST criterion that considers stable disease for changes < 20% (red arrow) (Fig. 3B). This absorbed dose separates Zone 1 and Zone 2. Above this absorbed dose, no lesions progessed (Zone 2), but in Zone 3 (MITV_SSTR_ close to 0%), the absorbed dose does not significantly improve the response [30]. Zone 1 (“unpredictable response zone”) corresponds to absorbed dose < threshold absorbed dose. Changes in MITV_SSTR_ values do not directly correlate with the absorbed dose and remain for some lesions above 20%, suggesting an increased SSTR^+^ tumour burden. In Zone 2 (“effective response zone”), MITV_SSTR_ increases less than 20% for all lesions (absorbed dose > threshold absorbed dose). Zone 3 ("unnecessary absorbed dose zone") reflects a decrease in MITV_SSTR_ values comparable to that observed in zone 2, indicating that the response might have reached a plateau (Fig. 3B).

#### Mileva’s study

In the study by Mileva et al*.*, 37 patients with NETs receiving a minimal lesion mean absorbed dose of 35 Gy from [^177^Lu]Lu-DOTA-TATE treatment (7.4 GBq) in the first cycle (~ 126 Gy after 4 cycles) exhibited a significantly longer PFS (48.1 vs. 26.2 months; HR, 0.37; 95% CI, 0.17–0.82; p = 0.02; *n* = 37 patients) [28]. This would place the threshold absorbed dose at ~ 120 Gy, which is probably reached after several cycles for some lesions, while it may never be reached for progressing lesions (size increase > 20% from baseline).

In all three studies, the lesion absorbed dose progressively decreased with each cycle, and the calculated absorbed doses were very heterogeneous (tens of Gy to ~ 400 Gy) [28–30]. In addition, the same absorbed dose may cause different effects in different tumour lesions, and conversely, lesions receiving different doses may show the same response [29]. Therefore, besides the absorbed dose, additional parameters need to be taken into account when considering the response to a treatment with ionising radiation, including the tumour biology [35]. The interpretation of the correlation between absorbed dose and biological effect depends on where the absorbed dose value of the lesion is located on the “absorbed dose–response curve”, i.e., in which zone (zone 1, 2 or 3, see Fig. 2B).

## Clinical lessons from dosimetry: normal tissue complications

Normal tissue complications after TRT can result from the expression of the molecular targets of TRT outside the tumour, and from the unspecific retention of the radiopharmaceutical in healthy tissues. Complications include deterministic (e.g., haematological, kidney, salivary and lachrymal gland lesions) and stochastic effects (e.g., myelodysplasia). Because of its lower dose rate, TRT is expected to be less toxic per Gy for normal tissues than EBRT [36]. For example, 13.3 Gy (at 1 Gy.min^−1^) versus 34.2 Gy delivered at a continuous dose rate of 0.02 Gy.min^−1^ X-rays are required to observe pneumonitis in 50% of exposed rats [37]. However, normal tissues respond differentially to low dose rates (< 1 Gy.h^−1^) [21] and only late responding tissues with slower proliferation rate (e.g., kidneys) may be protected by decreasing the dose rate [16, 21]. Another important toxicity parameter is the role of the irradiated volume [21, 38] because normal tissues can recover and maintain their functionality (parallel response architecture) in non-irradiated areas or areas irradiated at low absorbed doses.

### Bone marrow toxicity and bone marrow absorbed doses

Bone marrow toxicity is generally the limiting criterion of TRT. Toxicity occurs within 4–6 weeks after therapy and can last several months. At early time-points, toxicity is due to the loss of peripheral blood cells and later toxicity is caused by death of bone marrow progenitors (particularly in the presence of bone disease [39]). Bone marrow is a hierarchical tissue (i.e., organised into successive FSU corresponding to stem cells, differentiating cells and differentiated cells) and is considered a fast proliferating (progenitors) and early responding tissue. In EBRT, bone marrow aplasia occurs with absorbed doses > 3 Gy and haematological syndrome leads to death in 50% of cases after whole body exposure to 4.5 Gy. When irradiation is not fatal, the hematopoietic stem cell level will return to normal progressively, but the process can take years. Moreover, in EBRT, dose fractionation is not expected to counterbalance cell killing [16]. On the contrary, increasing the time between the first and last cycle is beneficial, although it will also benefit the tumour [16].

Because of its low dose rate, we would expect these toxicities to occur at higher absorbed doses during TRT. Indeed, it was shown that 3 Gy delivered during TRT to bone marrow led to grade 3/4 haematological toxicity only in 5 of 32 patients (16%) after two additional cycles of [^177^Lu]Lu-DOTA-TATE [40].

In TRT, heterogeneous absorbed doses values are generally reported for bone marrow [34]. For example, 177–994 mGy/MBq (from bone surface) and 1–5 mGy/MBq (from blood) were determined for 100 kBq/kg per cycle of ^223^Ra [34, 41]. In the VISION study, treatment with [^177^Lu]Lu-PSMA-617 (6 cycles of 7.4 GBq) resulted in mean red marrow absorbed doses of 0.035 ± 0.02 Gy (range, 0.020–0.13 Gy) after the first cycle, 0.031 ± 0.007 Gy (range, 0.021–0.051 Gy) per cycle for cycles 2–6, and < 0.2 Gy for all six cycles combined [42]. This led to the following toxicities that occurred after the start of randomised treatment and up to 30 days after the last administration (*n* = 519 patients) [43]: anaemia grade ≥ 3 (12.9% vs. 4.9% in the standard of care [SOC] group), thrombocytopenia grade ≥ 3 (7.9% vs. 1% in SOC), lymphopenia grade ≥ 3 (7.8% vs. 0.5% in SOC) and leukopenia grade ≥ 3 (2.5% vs. 0.5% in SOC). Adverse events (all included) led to reduction of activities, interruption and discontinuation of the treatment in 1.9%, 7.9%, and 7.0% of the patients, respectively, and to death in 3.6% of them [43]. Similar findings were observed in [44].

### Threshold absorbed dose for bone marrow toxicity: example for [^177^Lu]Lu-DOTA-TATE

Bergsma et al*.* [45] obtained high bone marrow absorbed doses in 23 patients treated with 4 cycles of [^177^Lu]Lu-DOTA-TATE. After the last cycle, the bone marrow absorbed dose was ~ 0.067 Gy/GBq (i.e., about 0.49 Gy per cycle of 7.4 GBq or ~ 2 Gy for the 4 cycles). Similar bone marrow absorbed doses from [^177^Lu]Lu-DOTA-TATE were determined by other groups [46, 47]. In the study by Bergsma et al*.*, 34 (11%) of 320 patients treated with four cycles of 7.4 GBq [^177^Lu]Lu-DOTA-TATE developed grade 3/4 haematological toxicity that necessitated blood transfusion in 15/34 patients [45]. Myelodysplastic syndrome and acute leukaemia were observed in 1–2% of patients. The link between haematological toxicity and the bone marrow absorbed dose was suggested by some authors [45] (for a given biological endpoint, such as platelet count), but not by others [35].

**Fig. 4:**
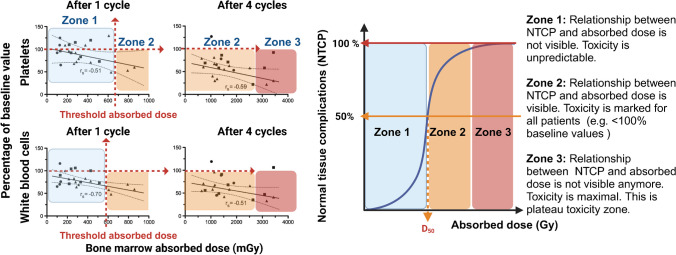
**A** Platelets and white blood cell counts expressed as percentages of the baseline values as a function of the bone marrow absorbed dose in 23 patients with NET after one or four cycles of [^177^Lu]Lu-DOTA-TATE (solid circles: group 1, 1.85 GBq, *n* = 4; solid squares: group 2, 3.70 GBq, *n* = 7; solid triangles: group 3, 7.40 GBq, *n* = 12) (figure adapted from [45]). The 100% of baseline values was arbitrarily chosen as the toxicity criteria for delineating Zone 1 and 2. Zone 1 corresponds to absorbed doses associated with values larger and smaller than 100% of the baseline value (unpredictable toxicity zone, absorbed dose < ~ 0.6–0.7 Gy) for both platelets and white blood cells. The “marked toxicity zone” corresponds to absorbed doses leading in all patients to values < 100% of the baseline value. This corresponds to absorbed doses between ~ 0.6–0.7 Gy and ~ 3 Gy. A zone 3 is proposed for absorbed doses > 3 Gy. It corresponds to the “plateau toxicity zone” where maximal toxicity is observed. B) Definition of the zones for NTCP

The authors reported the level of platelets and white blood cells as a function of the bone marrow absorbed doses [45]. As we did before for tumour lesions, we propose to delineate three zones in the Figure from this study (Fig. 4A) with an arbitrary biological criterion corresponding to 100% of the base value: the “unpredictable toxicity zone” is observed for absorbed doses < ~ 0.6 Gy and the *“*marked toxicity zone” at higher absorbed doses. It should be noted that this zone begins with the first cycle and is even more pronounced with the following cycles. We also propose a zone 3 (“plateau toxicity zone”), with an arbitrary biological criterion corresponding to 50% of the base value and likely to lead to grade 3 toxicities in some patients At this stage, complementary data from other forthcoming studies are needed to define the precise contour of these zones (Fig. 4B).

### Threshold absorbed dose for kidney toxicity

Kidneys are the second organ at risk due to the excretion of small-sized radiopharmaceuticals (< 75 KDa) in urine. High accumulation of PSMA- and SSTR-binding radioligands is, for example, observed in kidneys as a result of proximal tubular reabsorption of the radiopeptide, its retention in the renal interstitium [35], and for PSMA-binding radioligands, of PSMA-expression in kidneys [48]. However, nephrotoxicity of grade ≥ 3 does not seem to occur in most patients with normal renal function and treated with PSMA- or SSTR-targeted TRT [31]. As kidneys have a parallel histological organisation (i.e., functional subunits are arranged in parallel not in series like in bone marrow), the threshold absorbed dose for measuring 50% of kidney dysfunction depends on the volume of irradiated kidney. In EBRT, it is 8 Gy if 100% of the kidney volume is irradiated and increases to 27 Gy when 10% of the volume is irradiated [49]. This is in agreement with QUANTEC (Quantitative Analyses of Normal Tissue Effects in the Clinic) data show that when less than 20% of the kidney volume is exposed to 28 Gy only < 5% of patients will develop a clinically relevant kidney dysfunction [50].

However, such data are not available for TRT, and no threshold absorbed dose for a given level of toxicity is available. For [^177^Lu]Lu-PSMA-617, the kidney absorbed dose was below 20 Gy [42] with 0.43 ± 0.16 Gy/GBq in cycle 1 and 0.44 ± 0.21 Gy/GBq in cycles 2–6. Similarly, after 4 cycles of 7.4 GBq [^177^Lu]Lu-DOTA-TATE, the mean absorbed dose to the kidneys was 20.1 ± 4.9 Gy and only 3/323 (1%) patients developed (subacute) renal toxicity grade 2 (increase in serum creatinine > 1.5 – 3.0 times the baseline or upper limit of normal) [51]. No subacute grade 3 or 4 nephrotoxicity was observed and no significant nephrotoxicity was reported during the follow-up [51]. Importantly, the absorbed dose to kidneys after [^177^Lu]DOTA-TATE administration can be reduced by up to 50% through the simultaneous administration of positively charged amino acids (e.g., L-arginine and L-lysine) [52]. However, this approach does not work for [^177^Lu]Lu-PSMA-617.

### Salivary and lacrimal glands

Adverse effects are also observed in salivary and lacrimal glands exposed to [^177^Lu]Lu–DOTA-PSMA. Transient xerostomia (i.e., dry mouth) of grade 1–2 was observed in 60% of patients in a phase 2 trial and in 38.8% of patients in the VISION trial [3, 44]. Xerostomia is often permanent with [^225^Ac]Ac-PSMA alpha-particle TRT. Limiting salivary gland uptake by administering radiation protection and alleviating symptoms by stimulating salivary gland secretion have not been successful to date.

Absorbed doses to parotid glands (the largest salivary glands) are 0.1–1.9 Gy/GBq [^177^Lu]Lu-DOTA-PSMA617 cycle [32, 53]. Salivary glands are sensitive to radiation and at 10–15 Gy X-rays, saliva production is significantly reduced after the first week of therapy and can be completely abrogated by an absorbed dose of 40 Gy within 4 weeks [54]. Like for kidneys, due to the organisation of the salivary glands, the radiation tolerance dose depends on the volume irradiated. The total volume irradiation leading to atrophy/fibrosis is 60–70 Gy EBRT [38], but one-third capacity is sufficient for saliva production. Therefore, reducing the irradiated volume using short range particles is relevant, but the absorbed dose also must be reduced. It is likely that increasing treatment duration might have some benefits. Therefore, understanding the processes underlying the uptake of PSMA-targeting radioligands in the salivary glands is an important step to prevent or reduce xerostomia after PSMA-TRT [55], particularly with alpha emitters [56]. Likewise, PSMA expression in the lacrimal glands can expose these glands to an absorbed dose of ~ 80 Gy in a six-cycle TRT-regimen. Dry eyes is another reported side effect [57]. In addition, a case report described visual disturbances, possibly caused by treatment with [^177^Lu]Lu-PSMA-617 [58].

## Mean absorbed dose alone is not enough

We have shown that the absorbed dose is the first parameter involved in TCP or NTCP. In theory, for absorbed doses below a given threshold, it is not possible to predict response or toxicity, while for absorbed doses above that threshold (considering an arbitrary biological criterion, e.g., lesion volumes do not progress beyond 20%), all lesions should respond or toxicity will be observed in all patients. This does not hide the fact that the same absorbed dose delivered to different lesions may induce different responses and that different absorbed doses administered to different lesions may induce the same response.

**Fig. 5:**
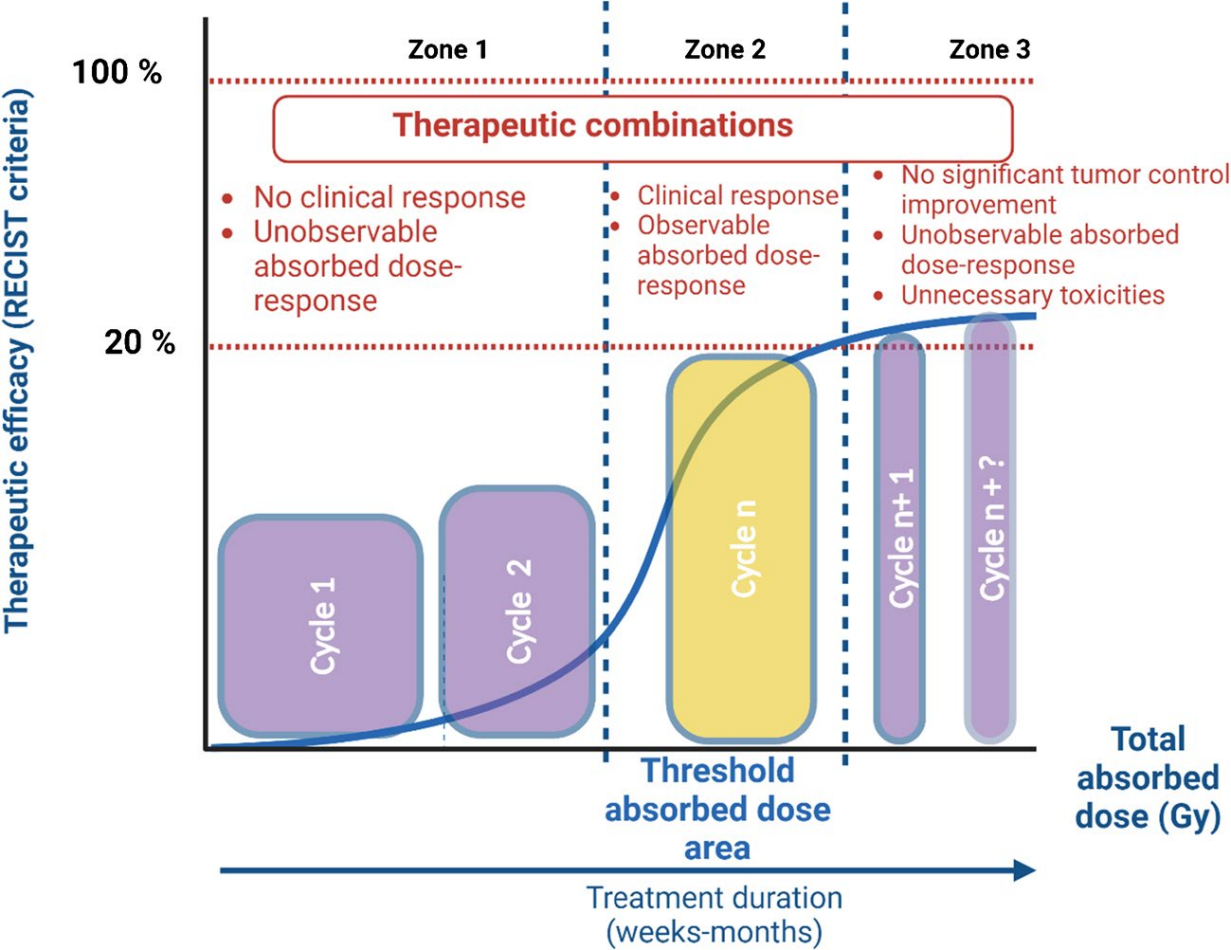
TCP during TRT. Influence of cycles to reach the threshold absorbed dose and of therapeutic combinations

Conversely to EBRT, one of the difficulties with TRT is that it is used for metastatic disease. This means that the threshold-absorbed dose will be reached for some of the patient's lesions, but not for others. To deal with this, it is possible to increase the number of cycles (e.g., from 4 to ≥ 6 cycles of [^177^Lu]Lu-DOTA-TATE or [^177^Lu]Lu-DOTA-PSMA617). By increasing this number, the total absorbed dose delivered to each lesion will increase and thereby, the number of lesions irradiated above threshold absorbed dose. However, this will inevitably be accompanied by greater toxicity and the threshold absorbed dose will anyways not be reached for certain lesions. Furthermore, in zone 3, the absorbed dose loses its effectiveness and the gain for the patient is very limited. The reason why lesions receiving higher absorbed doses do not respond better to treatment remains unexplained. If such response plateaus were observed with a satisfactory TCP (90–100%), the situation would be acceptable. However, the actually achieved TCP values are not satisfactory because TRT does not cure in almost all cases, but only stabilises the disease. Therefore, we need to look for other options for improvement. This is where other parameters involved in the response need to be considered, such as intra- and inter-patient heterogeneity in target expression, anatomical disease location, (epi)genetics, DNA repair capacity, and role of the tumour microenvironment. Consideration of these parameters will make it possible to identify the most effective therapeutic combinations (Fig. 5). This will be the subject of part II of the EANM group's publication on this subject.

## Conclusion

In this first part of our analysis, we discussed how knowledge of radiobiology, often obtained by studying EBRT, but increasingly also in TRT, could provide useful information for the development of therapies with highest possible efficacy and low toxicity profiles. We conclude that there are still too few dosimetric data available, although they are essential for setting up treatments. Our analysis also highlighted the nature of the absorbed dose–response relationships that can be expected. Data indicate that below the threshold absorbed dose specific for a given TCP or NTCP, it is impossible to predict the tumour or healthy tissue response. On the other hand, above this threshold absorbed dose, the likelihood for a given biological response may be enhanced. However, the potential existence of a zone 3 (or plateau) indicates that even at increasingly higher mean and heterogeneous absorbed doses, this parameter is no longer sufficient to explain the absence of response to treatment. This observation also applies to zones 1 and 2, where a low proportion of lesions respond to treatment, but where the dose ranges absorbed are also too low. This indicates that a better understanding of the radiobiology of healthy tissues and tumours is needed. It is also for these zones in particular that therapeutic combinations will find their full rationale.

## Data Availability

This is a review article and all analysed data are available in the corresponding references.
